# The evolutionary history of the sucrose synthase gene family in higher plants

**DOI:** 10.1186/s12870-019-2181-4

**Published:** 2019-12-18

**Authors:** Xiaoyang Xu, Yongheng Yang, Chunxiao Liu, Yuming Sun, Ting Zhang, Menglan Hou, Suzhen Huang, Haiyan Yuan

**Affiliations:** 1 Jiangsu Key Laboratory for the Research and Utilization of Plant Resources, Institute of Botany, Jiangsu Province and Chinese Academy of Sciences, Nanjing, 210014 China; 2 Institute of Pomology, Jiangsu Academy of Agricultural Sciences/Jiangsu Key Laboratory for Horticultural Crop Genetic Improvement, Nanjing, 210014 China

**Keywords:** Sucrose synthase, Gene family, Evolution, Phylogeny, Expression pattern

## Abstract

**Background:**

Sucrose synthase (SUS) is widely considered a key enzyme participating in sucrose metabolism in higher plants and regarded as a biochemical marker for sink strength in crops. However, despite significant progress in characterizing the physiological functions of the SUS gene family, knowledge of the trajectory of evolutionary processes and significance of the family in higher plants remains incomplete.

**Results:**

In this study, we identified over 100 SUS genes in 19 plant species and reconstructed their phylogenies, presenting a potential framework of SUS gene family evolution in higher plants. Three anciently diverged SUS gene subfamilies (SUS I, II and III) were distinguished based on their phylogenetic relationships and unique intron/exon structures in angiosperms, and they were found to have evolved independently in monocots and dicots. Each subfamily of SUS genes exhibited distinct expression patterns in a wide range of plants, implying that their functional differentiation occurred before the divergence of monocots and dicots. Furthermore, SUS III genes evolved under relaxed purifying selection in dicots and displayed narrowed expression profiles. In addition, for all three subfamilies of SUS genes, the GT-B domain was more conserved than the “regulatory” domain.

**Conclusions:**

The present study reveals the evolution of the SUS gene family in higher plants and provides new insights into the evolutionary conservation and functional divergence of angiosperm SUS genes.

## Background

Sucrose is the main end product of photosynthesis in higher plants and is exported from source leaves to sink organs. Sucrose catabolism in plants is one of the largest metabolic fluxes in the world, and it plays critical roles in carbon resource allocation and sugar signalling initiation [1, 2]. Sucrose is hydrolysed either by invertase (INV) into glucose and fructose or by SUS, which catalyses the reversible conversion of sucrose and uridine diphosphate (UDP) to fructose and UDP-glucose [1–3]. There is compelling evidence for the role of SUS in facilitating the entry of carbon into the metabolism of nonphotosynthetic plant cells and in determining sink strength in crop species. For instance, the *rugosus4* (*rug4*) mutation in pea reduces seed mass and starch content [4], and the *shrunken1* (*sh1*) mutation in maize leads to a shrunken seed phenotype due to the disruption of endosperm cell wall integration [5]. Antisense inhibition of specific isoforms of SUS genes reduces fruit setting and the sucrose unloading capacity of young fruit in tomato [6], decreases starch accumulation in potato tubers [7], affects the biosynthesis of cellulose and starch in carrot [8], and represses fibre cell initiation, elongation, and seed development in cotton [9]. Overexpression of the potato *Sus4* gene increases the levels of starch, adenosine diphosphate (ADP)-glucose and UDP-glucose and total yield in potato [10], increases the levels of starch and ADP-glucose in maize seed endosperm [11], and reduces seed abortion and enhances fiber production in cotton [12].

SUS is encoded by a small multigene family in the higher plants examined to date. Studies on the SUS genes of individual species have revealed that structural conservation and expressional and functional divergence followed gene family evolution. The *Arabidopsis* SUS gene family contains only six SUS genes with different but partially overlapping expression profiles [13], and their roles have been investigated through corresponding knockout mutants [14]. *AtSUS1* and *AtSUS4* show significant induction under hypoxia, and double mutant of these two genes exhibits reduced growth rates in hydroponic culture [14]. *AtSUS5* and *AtSUS6* are expressed specifically in the phloem and have a specific function in callose synthesis [15]. Pea harbours an SUS gene family containing at least three divergent genes, namely, *Sus1*, *Sus2* and *Sus3*, and these three genes show distinct patterns of expression in different organs and during organ development. Of these genes, *Sus1* displays a constitutive expression pattern and is highly expressed in the developing seed, and a lack of *Sus1* activity in *rug4* mutant of pea cannot be compensated by *Sus2* and *Sus3* [16]. In other plants, such as cotton, poplar, citrus and grape, the tissue-specific and development-dependent expression patterns of different SUS genes imply that each SUS gene may have evolved specialized physiological functions [17–20]. However, whether the divergence of plant SUS genes in expression and function occurred after the emergence of specific species, or at least to some extent in the common ancestor of angiosperms, is still unknown.

Despite compelling advances in determining the physiological functions and regulatory mechanisms of SUS genes, our knowledge of the evolutionary processes of the SUS gene family in higher plants remains incomplete. Molecular genetic research has provided substantial insights into the physiological function of individual proteins, while evolutionary analysis will shed light on the origin and expansion history of the gene family and further provide new insights into functional implications from an evolutionary perspective. For several years, our understanding of the evolution of the SUS gene family in plants has been based on surveys of individual angiosperm species [17, 20, 21]. Therefore, a comprehensive evolutionary analysis at a larger scale is necessary to achieve a better understanding of the SUS gene evolution in higher plants. In the current work, sixteen angiosperm species and three gymnosperm, fern ally and bryophyte species were chosen to show how the current SUS genes evolved and diverged from ancestral angiosperm SUS lineages. We investigated the classification, gene duplication, structural features, selection pressures and expression profiles of plant SUS genes. These results will provide a fundamental reference for understanding the evolutionary history of SUS genes and how evolutionary divergence contributes to the functional diversity of SUS genes.

## Results

### Classification of SUS genes in angiosperms

**Fig. 1:**
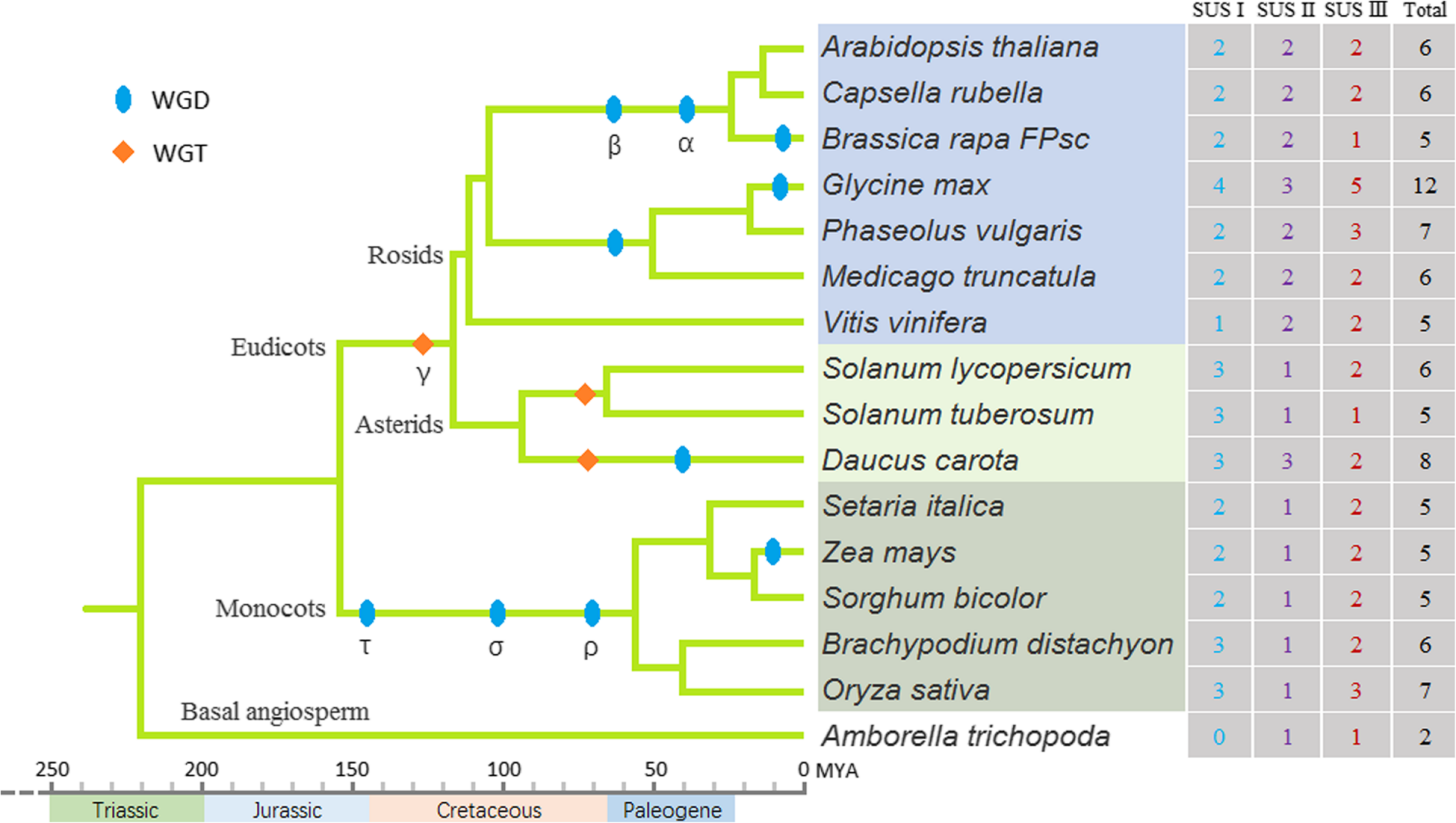
Identification and classification of SUS genes in 16 angiosperm species. The phylogenetic tree was constructed according to the AGP IV system [22]. Polyploidization events described in previous studies [23–26] are mapped onto the tree (blue ovals and orange diamonds). The total number of SUS genes and its classification in each plant are shown. WGD: Whole-genome duplication; WGT: Whole-genome triplication; MYA: Million years ago

A total of 96 SUS genes were identified in 16 angiosperm species including 10 dicot plants, 5 monocot plants and a basal angiosperm, *Amborella trichopoda*, using a hidden Markov model (HMM) and BLASTP searches (Fig. 1). Of all species surveyed, *Glycine max* possessed the greatest number of SUS genes (12), a number six-fold greater than that observed in *A. trichopoda* (2). Each of the remaining 14 species contained 5 to 8 SUS genes.

**Fig. 2:**
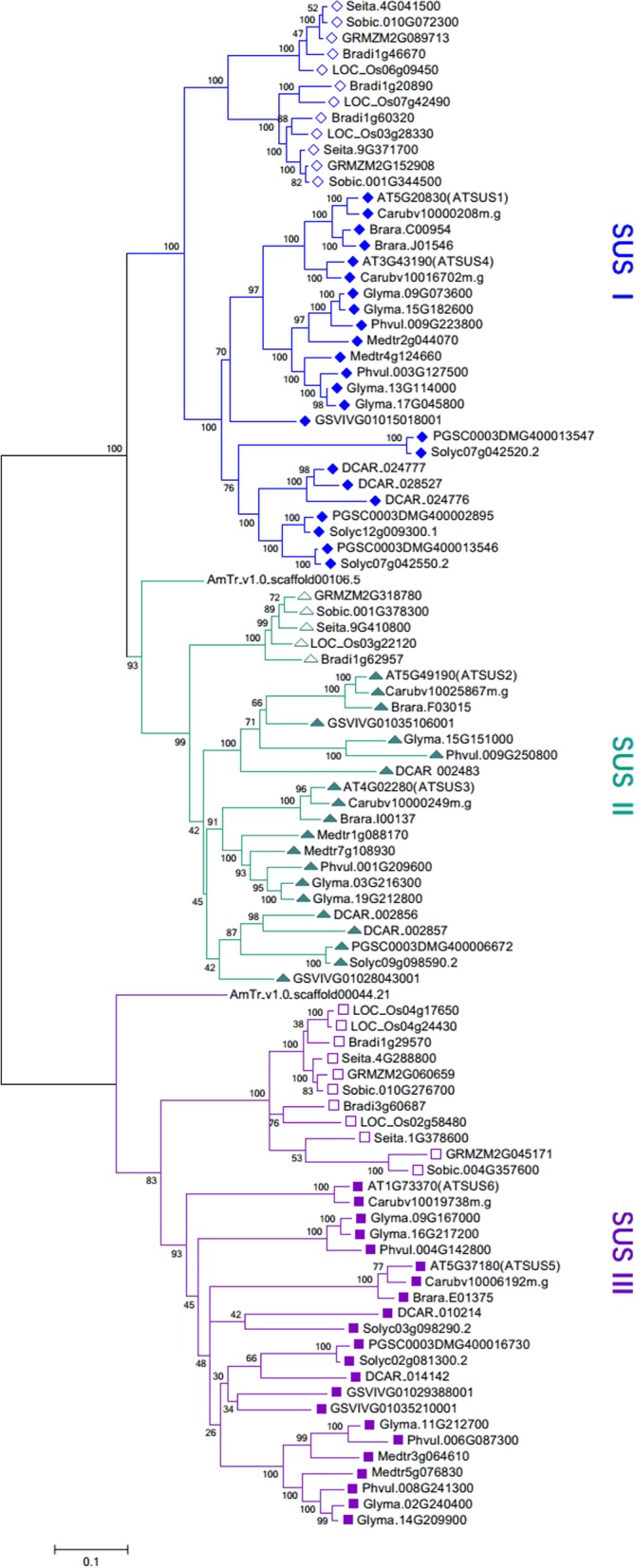
Phylogenetic analysis of 96 SUS genes from 16 angiosperm species. A Maximum Likelihood (ML) tree was constructed with MEGA 7.0 software [27] using amino acid sequences based on the Whelan And Goldman (WAG) model [28], the numbers on the branches represent the bootstrap supports. SUS genes from monocots are represented by hollow shapes, while those from dicots are represented by solid shapes

The 16 species surveyed in this study occupy important phylogenetic locations, as they include three major angiosperm lineages (monocots, asterids and rosids) and a basal angiosperm (Fig. 1). To trace the phylogenetic relationship of SUS genes in angiosperms, we constructed an unrooted phylogenetic tree of 96 SUS genes from these 16 species. The phylogenetic tree clearly classified the SUS gene family into three subfamilies named SUS I, II and III. Each subfamily further clustered into 3 groups: a monocot group, dicot group and basal angiosperm group, except for the SUS I subfamily, in which the basal angiosperm group was missing (Fig. 2). *AtSUS5* and *AtSUS6* contain a 3′ extension [13]. In the present study, almost all proteins of SUS III subfamily genes exhibited C-terminal extension (Additional file 1: Figure S1), indicating that SUS III genes of angiosperms may have evolved from a common ancestor.

**Fig. 3:**
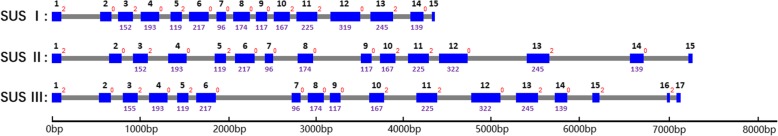
Proposed intron/exon structures of three ancestral SUS genes in angiosperms. Based on the intron/exon structures and the exon length of the 96 angiosperm SUS genes (Additional file 2: Figure S2; Additional file 6: Table S1), we reconstructed the intron/exon schematic structures of the three ancestral SUS genes in angiosperm. Blue boxes denote exons within coding regions, and the gray lines connecting them represent introns. We used the intron/exon structure of the Sobic.010G072300.1, AmTr_v1.0_scaffold00106.5, and AmTr_v1.0_scaffold00044.21 to represent the intron/exon schematic structure of angiosperm ancestral SUS I, II and III gene, respectively. Purple numbers under the blue boxes represent the sizes (bp) of corresponding conserved exons. Red numbers on the upper left of the gray lines represent the intron phase of corresponding introns. The length of the gray lines does not represent the actual sizes of the introns

Intron/exon arrangement is often regarded as an important parameter in gene phylogenies [29, 30]. To confirm the classification of SUS genes, we analysed the predicted intron/exon structure of the 96 SUS genes between the start and stop codons (Additional file 2: Figure S2). Based on the intron/exon arrangement and the exon length (Additional file 6: Table S1) of the 96 SUS genes that have been investigated, we reconstructed the proposed ancient intron/exons structure of SUS I, II and III genes in angiosperms (Fig. 3). The ancient SUS I and SUS II genes had 15 exons, while the ancient SUS III gene had 17 exons as it contained a 3′ extension. The length of the third to the fourteenth exons of the three ancient SUS genes was highly conserved. Intron loss was a common phenomenon in the descendants of the ancient SUS genes in angiosperms, especially in the dicot group of SUS I and SUS III genes. For the dicot group of the SUS I genes, introns were lost between the 5th and 6th exons and between the 12th and 13th exons. The introns were also lost between the 12th and 13th exons in the dicot group of SUS III genes. Intron loss occurred in only a small number of SUS II genes (Additional file 2: Figure S2). In general, SUS I, II and III genes had different intron/exon arrangements. Taken together, our phylogenetic and intron/exon structure analyses of the 96 SUS genes in angiosperms clearly show that there are three ancestral subfamilies of SUS genes predating the divergence of monocots and dicots.

### Evolutionary trajectory of SUS genes in higher plants

**Fig. 4:**
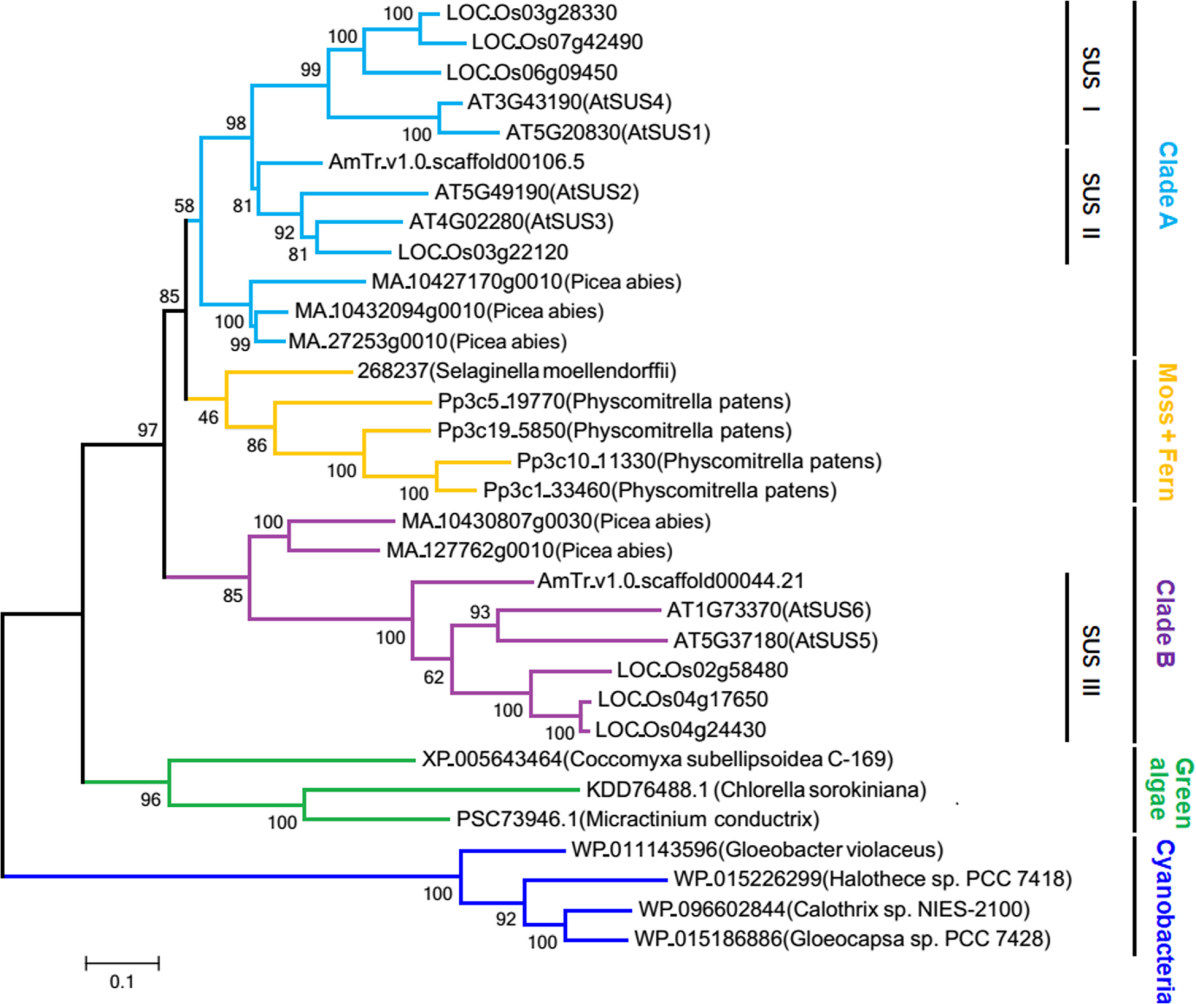
Phylogenetic analysis SUS genes from seed plants, bryophyte, fern, green algae and cyanobacteria. A ML tree was constructed with MEGA 7.0 software using amino acid sequences based on the WAG model, the numbers on the branches represent the bootstrap supports. SUS genes are from *A. thaliana*, *O. sativa*, *A. trichopoda*, *P. abies*, *S. moellendorffii*, *P. patens*, green algae and cyanobacteria

To trace the evolution of SUS genes in higher plants, we obtained 25 SUS homologous sequences from *Physcomitrella patens* (moss), *Selaginella moellendorffii* (fern), *Picea abies* (gymnosperm) and 3 angiosperm species (*A. trichopoda*, *Arabidopsis thaliana* and *Oryza sativa*). These sequences were then used to reconstruct a phylogenetic tree to infer the evolutionary trajectory of SUS genes with 7 SUS sequences from cyanobacteria and green algae as an outgroup. Two clades (clade A and clade B) of SUS genes were characterized in seed plants, both of which contained a gymnosperm branch and an angiosperm branch. SUS I and SUS II genes form the angiosperm branch of clade A, and SUS III genes form the angiosperm branch of clade B (Fig. 4). SUS genes of clade A are close to those of the bryophyte and pteridophyte branch in the phylogenetic tree (Fig. 4), and they also have similar intron/exon arrangements (Fig. 3; Additional file 3: Figure S3), indicating that clade A genes are more conserved than clade B genes. Previous research revealed an ancestral seed plant whole-genome duplication (WGD) event (ε) and an ancestral angiosperm WGD event (ζ) occurring shortly before the diversification of extant seed plants and extant angiosperms, respectively [31]. According to the phylogenetic relationship shown in Fig. 4, SUS I and SUS II genes may derive from the ancestral angiosperm WGD event, and clade A and clade B SUS genes may derive from the ancestral seed plant WGD event. Proteins of clade B SUS genes contained a C-terminal extension similar to that of bryophyte and pteridophyte SUS proteins (Additional file 4: Figure S4). However, clade B SUS genes exhibited two more 3′-end exons, which were not found in clade A, the bryophyte nor the pteridophyte (Fig. 3; Additional file 3: Figure S3), indicating that clade B SUS genes are a novel type of SUS gene in seed plants.

**Fig. 5:**
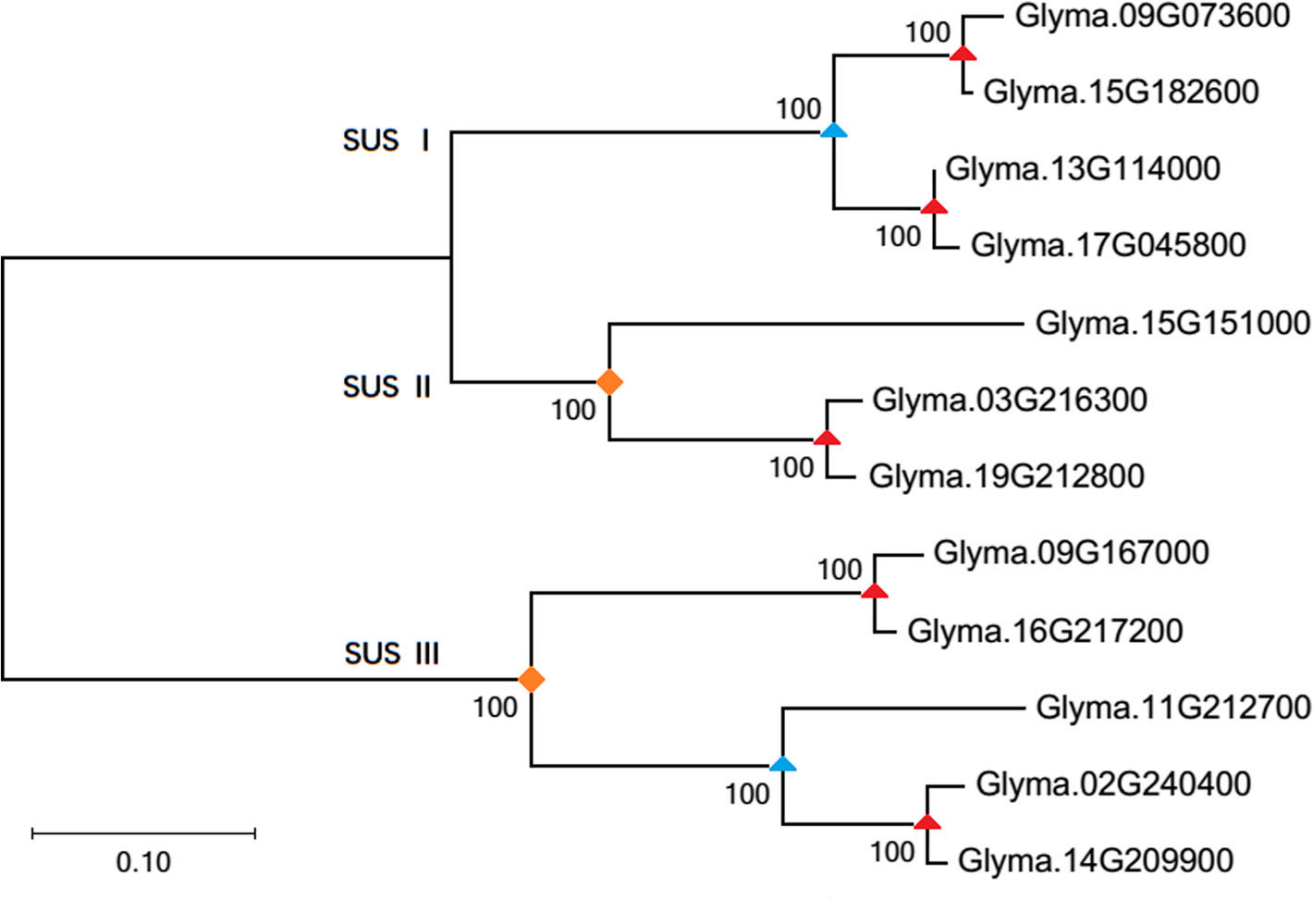
A phylogenetic tree of SUS genes of *G. max*. A ML tree was constructed with MEGA 7.0 software using amino acid sequences based on the WAG model, the numbers on the branches represent the bootstrap supports. SUS genes are from *G. max*. We calculate the *Ks* value of homologous gene pairs in each subfamily (Additional file 7: Table S2), and then interpret the replication nodes based on the *Ks* value. The common ancestor nodes marked by red triangles, represent the duplication event during the recent soybean lineage-specific WGD [25]. The common ancestor nodes marked by blue triangles, represent the duplication event during the ancient legume WGD [25]. The common ancestor nodes marked by orange diamonds, represent the triplication event during the core eudicots WGT [32, 33]

Furthermore, we inferred the expansion patterns of SUS genes in angiosperms. Tandem duplication rarely occurs in SUS genes, while the expansion of SUS genes in angiosperms is mainly through WGD. In monocots, a WGD event (τ) has been inferred that occurred after their divergence from the eudicot clade [32], and a whole-genome triplication event (γ) is probably shared by all core eudicots [33–35]. A series of successful WGDs has been inferred in association with the Cretaceous-Paleogene (K-Pg) boundary [36, 37]. There are also more recent WGDs that seem to have occurred independently in many different plant lineages (Fig. 1). Taking the SUS genes in soybean as an example, the ‘gamma’ event, the papilionoid lineage WGD event, and the soybean lineage-specific WGD event increased the number of SUS genes by 2, 2 and 5, respectively (Fig. 5; Additional file 7: Table S2). Because of the high retention ratio of duplicated SUS genes in the soybean-lineage-specific WGD event, the number of SUS genes in soybeans is almost twice that in other species (Fig. 1). Interestingly, no expansion was found in the SUS II genes in the five monocot plants we investigated; they each contained only one SUS II gene, which was the same as the number found in *A. trichopoda*.

In summary, there are two monophyletic clades of SUS genes in seed plants, and clade B SUS genes may be a novel clade as they contain two more 3′-end exons. In angiosperms, there are three subfamilies of SUS genes, and the expansion of SUS genes in angiosperms is mainly through WGD, although the SUS II genes in grass have not expanded.

### SUS III genes evolved under relaxed purifying selection in dicots

**Fig. 6:**
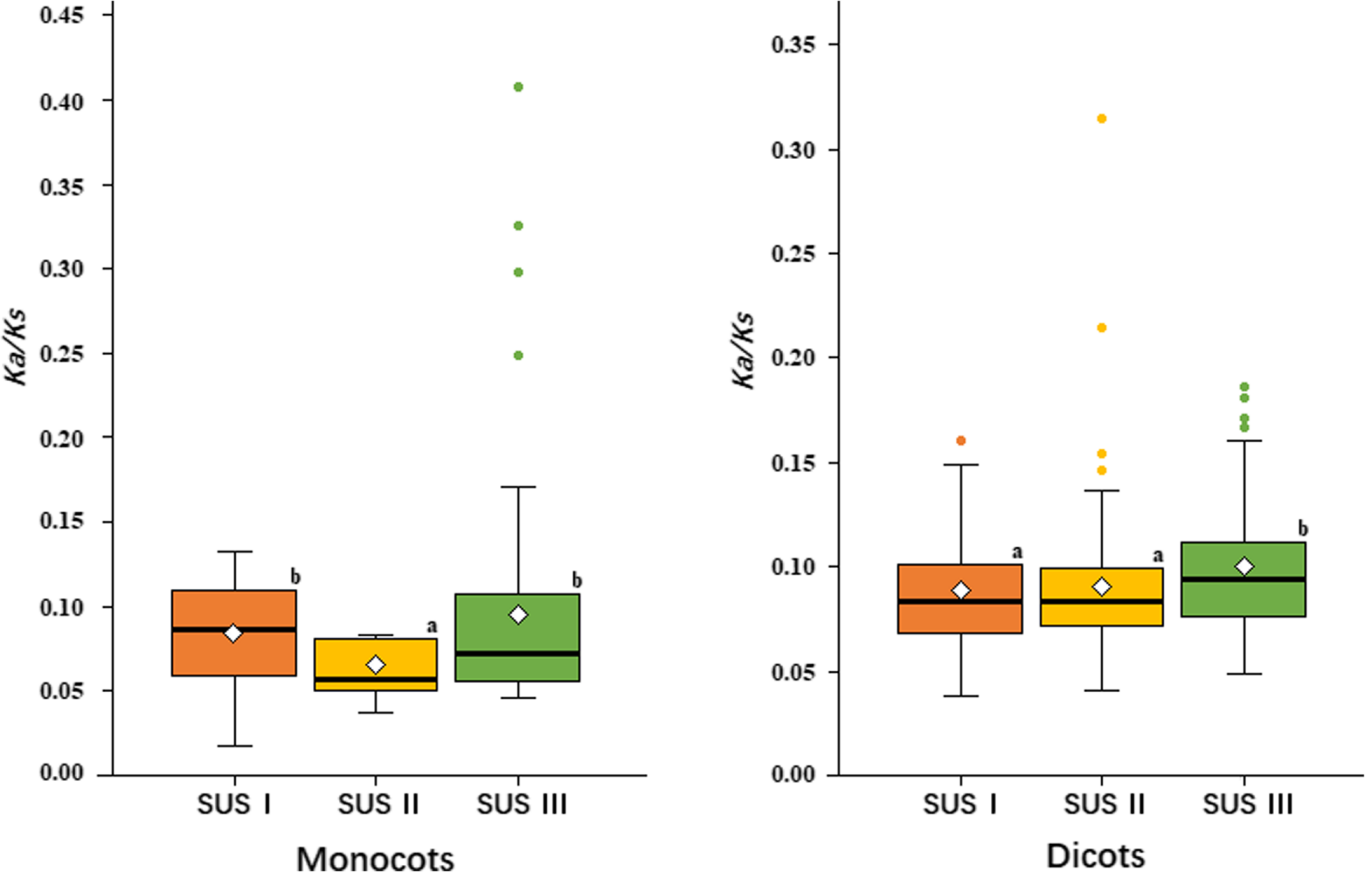
The overall ω values of three SUS subfamily genes in monocots and dicots. The top and bottom edges of the box indicate the upper and lower quartiles, the central black line show median value and the diamond show mean value. The whiskers extend 1.5 times of the interquartile range beyond the edges of the box. Different letters above the boxes indicate significant differences (one way-ANOVA and Dunnett’s T3 test; *P* < 0.05)

There is increasing evidence that different subfamilies from the same gene family may experience different purification selection pressures, form relatively fixed expression patterns and have different functions [38, 39]. To determine whether SUS genes are under different evolutionary constraints in angiosperms, the overall ratio of nonsynonymous substitutions per nonsynonymous site to synonymous substitutions per synonymous site (ω) values for the three subfamilies of SUS genes were calculated (Fig. 6; Additional file 8: Table S3). The overall ω value of SUS II genes (0.0630) was significantly lower than that of SUS I genes (0.0842) and SUS III genes (0.0962) in monocots, which indicated that the SUS II genes in monocots experienced strong purifying selection, probably because each of the five monocots we surveyed had only one SUS II gene (Fig. 1). In dicots, the overall ω value of SUS III genes (0.0990) was significantly higher than that of SUS I genes (0.0865) and SUS II (0.0889) genes, suggesting that SUS III genes were subjected to relaxed purifying selection and may have acquired new functions.

### Expression profiles of SUS genes

**Fig. 7:**
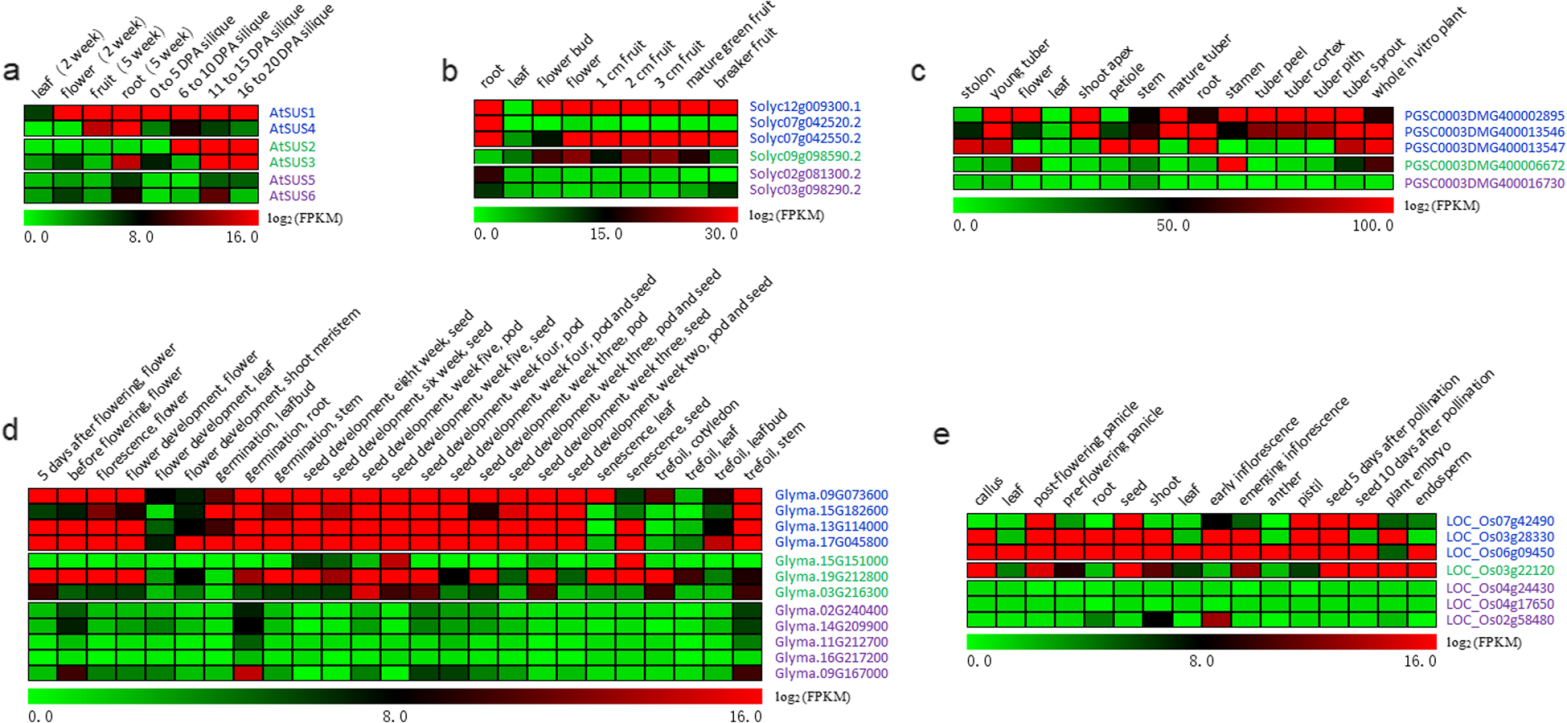
Expression profiles of SUS genes. **a** *A. thaliana*; **b** *S. lycopersicum*; **c** *S. tuberosum*; **d** *G. max*; **e** *O. sativa*. Transcript data from various organs and development stages of the five species were downloaded from Expression Atlas [40]. Gene names with different color markers indicate that these genes come from different SUS subfamilies. Blue: SUS I subfamily; green: SUS II subfamily; purple: SUS III subfamily. FPKM: Fragments per kilobase of exon model per million reads mapped

Furthermore, we investigated the expression profiles of three subfamilies of SUS genes in *A. thaliana*, *G. max*, *Solanum lycopersicum*, *Solanum tuberosum* and *O. sativa* from publicly available RNA-seq data (Fig. 7). In *Arabidopsis*, *AtSUS5* and *AtSUS6* from the SUS III subfamily were expressed only in specific tissues, while SUS I (*AtSUS1* and *AtSUS4*) and SUS II (*AtSUS2* and *AtSUS3*) genes showed broader expression than SUS III genes (Fig. 7a). A similar scenario was also found in four other plant species (tomato, potato, soybean and rice; Fig. 7b-c). These expression data revealed that SUS III genes exhibited more tissue-specific expression patterns than SUS I and SUS II genes. Furthermore, SUS I genes from the five species displayed constitutive expression in diverse vegetative and reproductive organs (Fig. 7).

The distinct expression patterns of SUS genes in dicots (Fig. 7) agree with the different selection pressures they experienced (Fig. 6). SUS III genes evolved under relaxed purifying selection in dicots and showed tissue-specific expression patterns, while SUS I genes, which experienced greater evolutionary constraints, showed broader expression patterns. Interestingly, although SUS III genes did not evolve under relaxed evolutionary constraints, in contrast to SUS I genes in monocots, SUS III genes from rice still exhibited tissue-specific expression patterns (Fig. 7e). These findings reveal the conserved functions of SUS I genes in maintaining cellular sucrose metabolism, and SUS III genes may acquire new functions in the evolution of angiosperms. A similar pattern was observed for another gene involved in sucrose metabolism. The neutral/alkaline invertase gene showed a broad or constitutive expression pattern and experienced greater evolutionary constraints than the acid invertase genes, which exhibited more tissue-specific expression patterns [38].

### CTD, EPBD and GT-B domains were subject to different selection pressures

**Fig. 8:**
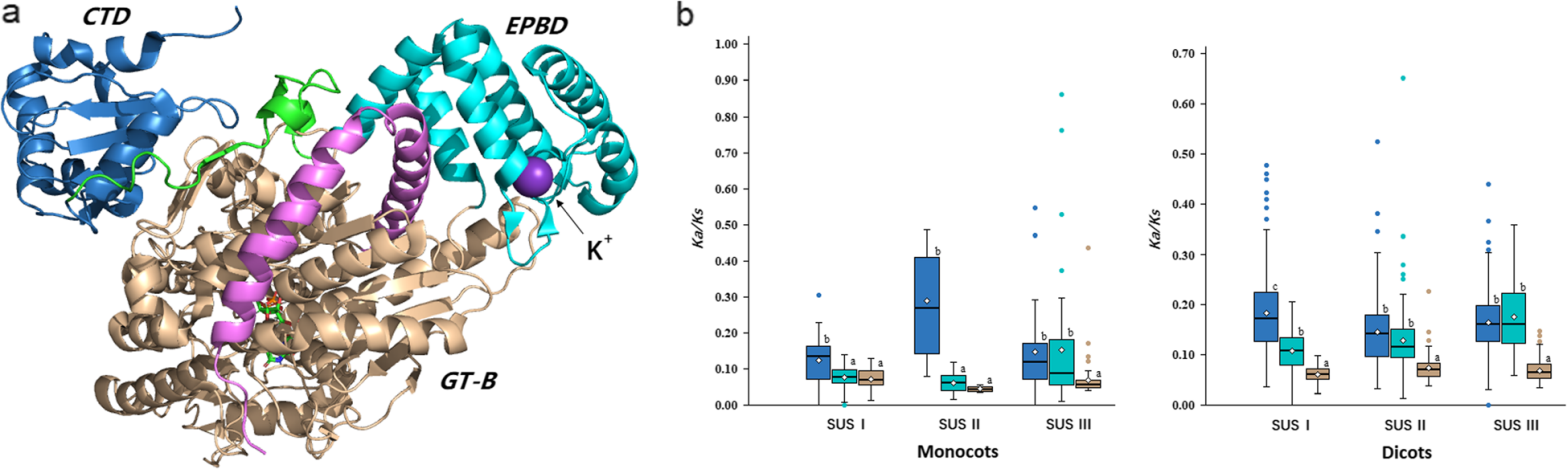
Structure and domain evolution of plant SUS. **a** Structure of AtSUS1 from *A.thaliana* (PDB code 3S27) [41]. Structural domains are highlighted in different colors: the CTD is colored blue; the EPBD is cyan; the linker between the CTD and EPBD is green, the GT-B domains are wheat, and the C-terminal extension is pink; **b** The overall ω values of CTD (blue), EPBD (cyan), and GT-B (wheat) domains of three SUS gene subfamilies in monocots and dicots. The top and bottom edges of the box indicate the upper and lower quartiles, the central black line show median value and the diamond show mean value. The whiskers extend 1.5 times of the interquartile range beyond the edges of the box. Different letters above the boxes indicate significant differences (one way-ANOVA and Dunnett’s T3 test; P < 0.05)

The plant SUS polypeptide chain consists of a cellular targeting domain (CTD), an early nodulin 40 (ENOD40) peptide-binding domain (EPBD), a typical GT-B domain, and a C-terminal (Fig. 8a) [41, 42]. The N-terminal “regulatory” domain, including the CTD and EPBD, is involved in cellular targeting, and the GT-B domain is involved in the glycosyl transfer reaction. The general kinetic properties of all six SUS genes in *Arabidopsis*, which rely on the GT-B domain, are closely related to each other [14]. Depending on the metabolic environment, SUS alters its cellular location from the cytosol to sites of cellulose, callose, and starch biosynthesis by its interactions with various organelle membranes [43–45] and cytoskeletal actin [46] through its “regulatory” domain [41]. The CTD, EPBD and GT-B domain of SUS exhibit different functions, and these domains may also experience different selection pressures. We calculated the ω values of three subfamilies of SUS genes in monocots and dicots. To our surprise, the ω values of the GT-B domains of all three subfamilies of SUS genes are lower than those of the “regulatory” domains (Fig. 8b-c; Additional file 9: Table S4). The active sites in the GT-B domains are almost identical, both within and among the three subfamilies (Additional file 5: Figure S5). Therefore, the GT-B domains are more conserved and are subjected to more evolutionary constraints than the “regulatory” domains.

## Discussion

Sucrose is found in a wide range of organisms including cyanobacteria, unicellular algae and especially higher plants. It is usually synthesized in cyanobacteria under salt or osmotic stress and is believed to help maintain osmotic balance [47, 48]. However, in most higher plants, sucrose is the main end product of photosynthesis, and sucrose metabolism plays pivotal roles in the allocation of carbon resources and in the initiation of sugar signalling [1, 2]. Sucrose is cleaved either by SUS into UDP-glucose and fructose or by INV into glucose and fructose [49]. The relationship between evolutionary steps and the functional implications of three types of INV have been elucidated by Wan et al. [38]. Given that the evolutionary history of SUS genes in higher plants remains fragmented and elusive, a comprehensive understanding of their evolutionary trajectory, structural features, expression profiles, and functional significance will be valuable for improving crop yield by optimizing carbon resource allocation.

### Origin, evolution and classification of plant SUS genes

The SUS gene might have originated in proteobacteria or a common ancestor of proteobacteria and cyanobacteria, and plants may have inherited it from cyanobacteria [48]. Benefiting from the whole-genome sequencing of various plant species, a large number of SUS genes have been identified through comparative genome approaches, which can be used to investigate the origin, evolution and classification of SUS genes in plants. In our present study, the phylogenetic analysis showed that SUS genes from plants formed a monophyletic group, suggesting that all plant SUS genes might have originated from a common ancestor [48] (Fig. 4). WGD, or polyploidy, which is often followed by substantial gene loss and diploidization, is a common phenomenon in plants [31]. The retained duplicated genes not only provide the genetic material necessary for biological innovation but also give rise to the diversity of plant homologous genes. SUS genes from seed plants formed two monophyletic clades (clade A and clade B) (Fig. 4), probably because the ancestor of seed plants experienced a WGD event [31], and the duplicated SUS gene copy was retained. Moreover, two subfamilies of angiosperm SUS genes in clade A (SUS I and SUS II) (Fig. 4) might stem from the ancestral angiosperm WGD event [31]. Plant SUS genes have historically been divided into three major subfamilies based on their phylogenetic relationship (Sus1, Sus A and New Group/NG) [13, 50]. The phylogenetic analysis of angiosperm SUS genes in our research is consistent with this classification, and we renamed them SUS I, II and III, respectively (Fig. 2). Furthermore, clade A genes are closer to the original type of plant SUS gene than are clade B genes, as the former genes clustered with the SUS genes of the bryophyte and pteridophyte in the phylogenetic tree (Fig. 4) and have ancient intron/exon structures similar to those of the bryophyte and pteridophyte SUS genes (Fig. 3; Additional file 3: Figure S3). In contrast, clade B genes may be a new type that appeared in seed plants.

Each subfamily of angiosperm SUS genes consisted of at least two independent groups, i.e., a monocot group and a dicot group (Fig. 2), which was consistent with the classification of 55 SUS genes in angiosperms [51]. In other studies involving the classification of angiosperm SUS genes, SUS II genes were also composed of the genes from monocots and dicots; however, genes from monocots and dicots did not group independently [17, 19], probably because the number of monocot SUS genes used was relatively small. Our results support the view that the SUS genes from monocots and dicots in each subfamily evolved independently. After the split of monocots and dicots, their ancestors underwent specific WGD events [32, 33, 35, 52]. Many species emerged during subsequent evolution and experienced lineage-specific WGD events [36]. WGD events and subsequent retention and loss of specific SUS genes led to different evolutionary trajectories of SUS genes in different species [17, 18, 20].

Intron/exon structures, to a certain extent, allow us to predict the possible origin and relationships of SUS genes [13, 50]. In our study, the first 14 exons of the three ancient SUS genes of angiosperms were highly conserved (Fig. 3), further suggesting that all three subfamilies of SUS genes may be derived from a common ancestor [48]. The most obvious difference in intron/exon structures among the three SUS subfamilies is that most SUS III genes have two more exons at the 3′ end (Additional file 2: Figure S2), which are not found in bryophyte and pteridophyte SUS genes (Additional file 3: Figure S3). However, the function of the 3′ extension in SUS III genes remains unknown. Each subfamily of SUS genes had different degrees of intron loss, and the intron loss between the 12th and 13th exons was conserved in dicots of both SUS I and III genes (Additional file 2: Figure S2) [13, 50]. The evolutionary and functional significance of intron loss in SUS genes requires further research.

### SUS I genes may play critical roles in sucrose metabolism in an O_2_-deficient environment

Sucrose metabolism is vital to multicellular plants and is degraded by either SUS or INV; however, the precise roles of these enzymes in specific plants remain largely unknown [2, 15]. Both SUS and INV appear as multiple, distinct isoforms. The cytoplasmic INV (CIN) genes are ancient and may play pivotal roles in maintaining cytosolic sugar homeostasis and cellular functions. The cell wall INV (CWIN) and vacuolar INV (VIN) genes are subject to relaxed purifying selection pressure, and CWIN genes have coevolved with vascular plants, probably as a functional component of phloem unloading [38]. SUS genes can be clearly divided into three subfamilies in angiosperms (Fig. 2) [13, 20]; however, the precise functions of each subfamily have not been elucidated. We speculate that each subfamily of SUS genes may have different functions.

Conversion of sucrose to hexose phosphates via SUS requires only half the adenosine triphosphate (ATP) needed for conversion via INV, and the SUS route is thought to be more effective than the INV route in an O_2_-deficient environment, where ATP synthesis may be limited [1, 14, 15]. The induction of some SUS genes by hypoxia or anoxia is a widespread phenomenon in both monocot and dicot species. The expression of *Sus1* and *Sh1* in maize, *Ss1* in wheat, and *Susy∗Dc1* in carrot is induced or enhanced under hypoxic or anoxic conditions [53–55], and all these genes originate from the SUS I gene subfamily. Likewise, in *A. thaliana*, transcript levels increase for *AtSUS1* and *AtSUS4* but not other SUS genes under hypoxia (Additional file 10: Table S5) [13, 56], and the double mutant of these two genes shows marked growth retardation under hypoxia [14]. Antisense suppression of a cucumber SUS I gene (*CsSUS3*) reduces hypoxic stress tolerance [57]. Consistently, some SUS genes have long been considered a biochemical marker for sink strength, especially in metabolically highly active or bulky organs where the endogenous oxygen level may be low. For instance, mutation of maize lacking either *Sus1* or *Sh1* leads to reduced starch content [5], whereas antisense inhibition of specific SUS genes drastically reduces starch accumulation in potato tubers [7], and represses fibre elongation and seed development in cotton [9]. Furthermore, we investigated the SUS genes associated with sink strength and found that almost all of these SUS genes derived from SUS I (Table 1). Accordingly, we speculate that SUS I genes but not SUS II and III genes are responsible for sucrose conversion in an O_2_-deficient environment. Consistent with this view, haplotype association revealed that two SUS I genes (*TaSus1* and *TaSus2*) from wheat were associated with thousand kernel weight, which mainly depends on the rate and amount of starch synthesis [58, 59]. The SUS I genes showed constitutive expression in diverse vegetative and reproductive organs (Fig. 7), and their roles in sucrose metabolism could be replaced by INV genes under normal oxygen levels [14]. However, in the case of insufficient oxygen content, SUS I genes are irreplaceable.

**Table 1:** SUS genes associated with sink strength

	Species	SUS gene	Locus	Subfamily	Functional or phenotypic description	Reference
Mutant	Maize	*ZmSus1* (L22296)	GRMZM2G152908	SUS I	*ZmSus1* product is needed mainly for generating precursors for starch biosynthesis.	Chourey et al.,1998
	Maize	*ZmSus2* (X02400)	GRMZM2G089713	SUS I	*ZmSus2* product performs a critical role in providing the substrate for cellulose biosynthesis.	Chourey et al.,1998
	Bea	*Sus1*(AJ012080)		SUS I	Pea mutants (*rug4*) lacking an isoform (Sus1) very similar to SUS1 and SUS4 of Arabidopsis have reduced seed mass and starch content.	Craig et al., 1999; Barratt et al., 2001
Antisense repression	Potato	*StSus4* (M18745)	PGSC0003DMG400002895	SUS I	Antisense inhibition of the main isoform SUS (*Sus4*) in potato tubers drastically reduces starch accumulation.	Zrenner et al.,1995
	Tomato	*SlSus1* (L19762)	Solyc12g009300.1	SUS I	Antisense inhibition of tomato fruit SUS (*SlSus1*) decreases fruit setting and the sucrose unloading capacity of young fruit.	D’Aoust et al., 1999
	Cotton	*GhSus1* (U73588)	Gohir.A05G036000	SUS I	Suppression of SUS gene expression represses cotton fiber cell initiation, elongation, and seed development.	Ruan et al., 2003
	Carota	SUS gene (X75332)	DCAR_028527	SUS I	Antisense repression of SUS affects the size of carrot plants and the biosynthesis of cellulose and starch.	Tang et al., 1999
Overexpression	Potato	*StSus4* (M18745), potato	PGSC0003DMG400002895	SUS I	Enhancing SUS activity in potato tubers results in increased levels of starch, ADP-glucose and UDP-glucose and total yield.	Baroja-Fernández et al., 2009
	Maize	*StSus4* (M18745), potato	PGSC0003DMG400002895	SUS I	Enhancing SUS activity results in increased levels of starch and ADP-glucose in maize seed endosperms.	Li et al., 2013
	Cotton	*StSus4* (M18745), potato	PGSC0003DMG400002895	SUS I	Overexpression of a potato SUS gene in cotton accelerates leaf expansion, reduces seed abortion, and enhances fiber production.	Xu et al.*,* 2012
	Poplar	*GhSus1* (U73588), cotton	Gohir.A05G036000	SUS I	SUS affects carbon partitioning to increase cellulose production.	Coleman et al., 2009
	Cotton	*GhSusA1*(HQ702185), cotton	Gohir.D08G139000	SUS II	Overexpression of *GhSusA1* increases plant biomass and improves cotton fiber yield and quality.	Jiang et al., 2012

SUS II and SUS I genes have similar ancient intron/exon structures and may stem from the ancestral angiosperm WGD event. These two subfamilies SUS genes showed different expression profiles (Fig. 7) [13], indicating that their functions may have undergone a certain degree of differentiation. For example, two SUS II genes from *Arabidopsis*, namely, *AtSUS2* and *AtSUS3*, are not induced in response to O_2_ deficiency, and the double mutant of these two genes is not obviously different from the wild-type (WT) control, although these two genes are strongly expressed in seeds [13, 14]. Jiang et al. [60] reported a cotton SUS II gene (Table 1), *GhSusA1*, which is closely associated with productivity as a key regulator of sink strength, indicating that some SUS II genes may have functional overlap with the SUS I gene in specific plants.

### SUS III genes exhibit a narrow expression profile, although their functions remain unknown

SUS III genes have intron/exon structures differing from those of SUS I and SUS II genes (Additional file 2: Figure S2), exhibit a narrow expression profile (Fig. 7), and are subject to relaxed purifying selection pressure in dicots (Fig. 6), suggesting that SUS III genes may have functions different from those of SUS I and SUS II genes. Wan et al. [38] reported that CWIN genes, which emerged in higher plants and show tissue-specific expression patterns, likely coevolved with the vascular development of higher plants. Two *Arabidopsis* SUS III genes, namely, *AtSUS5* and *AtSUS6*, are expressed only in specific tissues and organs (Fig. 6), and the proteins encoded by these two genes are present specifically in the phloem [15]. A double mutant of these two genes shows a thinner callose layer lining the pores of sieve plates than did WT plants. These two SUS III genes are considered to be involved in callose formation in the sieve plate [15]. Thus, we propose that SUS III genes may also be involved in the vascular development of higher plants.

### Evolutionary conservation and divergence of plant SUS genes

As discussed above, all plant SUS genes may have evolved from a common ancestor. In the plant species examined to date, SUS is encoded by a small multigene family. Comparative screening of the intron/exon structures of three subfamilies of SUS genes indeed revealed that the number and position of introns are highly conserved in angiosperms, although some introns were lost in specific SUS genes (Fig. 3; Additional file 2: Figure S2). Furthermore, all 16 active sites in the GT-B domain of AtSUS1 are almost identical among angiosperm SUS genes [41] (Additional file 5: Figure S5), and the GT-B domain has undergone strong purifying selection (Fig. 8). In addition, the isoforms of SUS from different subfamilies in *Arabidopsis* have similar kinetic properties [14]. All these results suggest that the SUS gene is structurally and functionally conserved in plants. Therefore, we speculate that different SUS genes may fulfil similar functions in different cell types or organelles at different developmental stages or under different stress conditions. Our analysis showed that three subfamilies of angiosperm SUS genes displayed distinct expression profiles, and these expression profiles may have been formed before the divergence of monocots and dicots. In general, homologous or duplicated SUS genes derived from WGD events within each subfamily inherited the expression patterns of their ancestors (Fig. 7). Moreover, according to the metabolic environment, SUS changes its cellular location to take part in the biosynthesis of cellulose, callose, and starch through its interactions with various organelle membranes and cytoskeletal actin [41]. The specificity of spatiotemporal expression and the variability of protein subcellular localization contribute to the functional diversity of SUS genes. Identifying the function of individual SUS genes is challenging not only because of the functional redundancy among duplicated SUS genes [14], but also because the VIN gene can partially functionally replace the SUS gene [15].

## Conclusions

The angiosperm SUS gene family can be divided into three subfamilies (SUS I, II and III) based on their phylogenetic relationships and unique intron/exon structures, and they were found to have evolved independently in monocots and dicots. Each subfamily of SUS genes exhibited distinct expression patterns in a wide range of plants, and SUS III genes evolved under relaxed purifying selection in dicots and displayed narrowed expression profiles. This work should provide a foundation for understanding the evolutionary history of SUS genes and how evolutionary divergence contributes to the functional diversity of SUS genes.

## Materials and methods

### Data collection

The genomic sequences, annotations and gene models of *A. thaliana*, *Capsella rubella*, *Brassica rapa*, *G. max*, *Pinguicula vulgaris*, *Medicago truncatula*, *Vitis vinifera*, *S. lycopersicum*, *S. tuberosum*, *Daucus carota*, *Setaria italica*, *Zea mays*, *Sorghum bicolor*, *Brachypodium distachyon*, *O. sativa*, *A. trichopoda*, *S. moellendorffii*, *P. patens*, and *Coccomyxa subellipsoidea* were collected from Phytozome (https://phytozome.jgi.doe.gov/pz/portal.html). Data for *P. abies* were downloaded from ConGenIE (http://congenie.org/). The tetrameric structure of AtSUS1 from *A. thaliana* was obtained from the Research Collaboratory for Structural Bioinformatics (RCSB) Protein Data Bank (PDB code 3S27).

### Identification of SUS genes

We combined an HMM and BLASTP searches to identify putative SUS genes in the 19 species. First, the HMM profiles of the SUS domain (PF00862) and glycosyl transferase group 1 domain (PF00534) were obtained from the Pfam website (http://pfam.xfam.org/), and these two HMM profiles were then employed as queries to identify all possible SUS genes using HMMER (V3.0) software. Second, the amino acid sequences of the six *AtSUS* genes [13] and seven *OsSUS* genes [61] were used to run a BLASTP search against all protein sequences in each species, with the threshold expectation value set to 1E-10. All hits obtained from HMM and BLASTP searches were merged together, and the redundant hits were removed. Finally, all candidate sequences were further subjected to online Pfam analysis (http://pfam.xfam.org/) to further confirm that they had both a SUS domain and glycosyl transferase group 1 domain. The protein sequences lacking the SUS domain or the glycosyl transferase group 1 domain were removed.

### Phylogenetic and gene structural analysis

Amino acid sequences were aligned using ClustalW, and gaps and poorly aligned sections were manually removed. Phylogenetic tree reconstruction was performed with the maximum likelihood (ML) approach using the aligned amino acid sequences in MEGA v7.0. The parameters were as follows: model, WAG; bootstraps, 1000 replicates; and gaps/missing data, partial deletion. The structure of the SUS genes was parsed from general feature format (GFF) files, and diagrams of the intron/exon structures were drawn using the online program Plant Intron Exon Comparison and Evolution (PIECE) (https://wheat.pw.usda.gov/piece/GSDraw.php).

### Estimation of *K*_*a*_/*K*_*S*_ ratios

The codon sequences of homologous gene pairs were aligned using ClustalW based on the amino acid sequences. The ratio of nonsynonymous substitutions per nonsynonymous site (*K*_*a*_) to synonymous substitutions per synonymous site (*K*_*S*_) (*ω* value) was calculated using DnaSP version 5. Saturation effects were avoided by discarding the gene pairs for which *K*_*S*_ > 2. The *ω* value is commonly considered a measure of selection at the protein level, with values of *ω* > 1, =1 and < 1 indicating positive selection, neutral evolution and negative or purifying selection, respectively.

## Supplementary information

**Additional file 1:** **Figure S1.** C-terminal amino acid alignment of SUS genes.

**Additional file 2:** **Figure S2.** Intron/exon structural organization of angiosperms SUS genes. Blue boxes denote exons within coding regions, and the gray lines connecting them represent introns. Boxes of other colors represent fused exons, and the numbers above them indicate which exons are fused. Red boxes indicate the splitting of the exons. Due to the complexity of the 3′ end of the SUS III subfamily genes, we do not show the fusion of their exons.

**Additional file 3:** **Figure S3.** Intron/exon structural organization of SUS genes from *P. patens *and *S. moellendorffii*. Blue boxes denote exons within coding regions, and the gray lines connecting them represent introns. Red numbers on the upper left of the gray lines represent the intron phase of corresponding introns. Boxes of other colors represent fused exons, and the numbers above them indicate which exons are fused.

**Additional file 4:** **Figure S4.** C-terminal amino acid alignment of SUS genes from *A. thaliana*, *O. sativa*, *P. patens *and *S. moellendorffii.*

**Additional file 5:** **Figure S5.** Amino acid alignment of GT-B domain of SUS genes. The 16 active sites (His-287, Gly-302, Gly-303, Gln-304, Arg-382, His-438, Met-578, Arg-580, Gln-648, Asn-654, Glu-675, Phe-677, Gly-678, Leu-679, Thr-680, Glu-683) identified in the GT-B domain of AtSUS1 were colored in orange.

**Additional file 6:** **Table S1.** Exon length (bp) statistics of angiosperms SUS genes.

**Additional file 7:** **Table S2.** *Ks *values of SUS gene pairs within subfamily of *G. max*.

**Additional file 8:** **Table S3.** Estimates of *Ka/Ks *(ω) values of SUS genes.

**Additional file 9:** **Table S4.** Estimates of *Ka/Ks *(ω) values of three domains of SUS genes.

**Additional file 10:** **Table S5.** Expression patterns of *Arabidopsis *SUS genes under hypoxia. The expression data of six SUS genes in *Arabidopsis *comes from Gene Expression Omnibus (GEO) DataSets (GSE119327). We analyzed the expression of six SUS genes in *Arabidopsis *under hypoxia and found that only *AtSUS1 *and *AtSUS4 *were induced by hypoxia. adj. P.Val: *P*-value after adjustment for multiple testing. logFC: Log2-fold change between two experimental conditions.

## Data Availability

The datasets supporting the conclusions of this manuscript are included within the article and its additional files.

## Abbreviations

ADP: Adenosine diphosphate
ATP: Adenosine triphosphate
CIN: Cytoplasmic INV
CTD: Cellular targeting domain
CWIN: Cell wall INV
ENOD40: Early nodulin 40
EPBD: ENOD40 peptide-binding domain
FPKM: Fragments per kilobase of exon model per million reads mapped
HMM: Hidden Markov model
INV: Invertase
ML: Maximum likelihood
MYA: Million years ago
PDB: Protein Data Bank
SUS: Sucrose synthase
UDP: Uridine diphosphate
VIN: Vacuolar INV
WAG: Whelan And Goldman
WGD: Whole-genome duplication
WGT: Whole-genome triplication
WT: Wild-type
