# Supplementary figures and images for: The evolutionary history of the sucrose synthase gene family in higher plants
Source: BMC Plant Biol. 2019 Dec 18;19:566. doi: 10.1186/s12870-019-2181-4 (PMC6921546; doi:10.1186/s12870-019-2181-4)

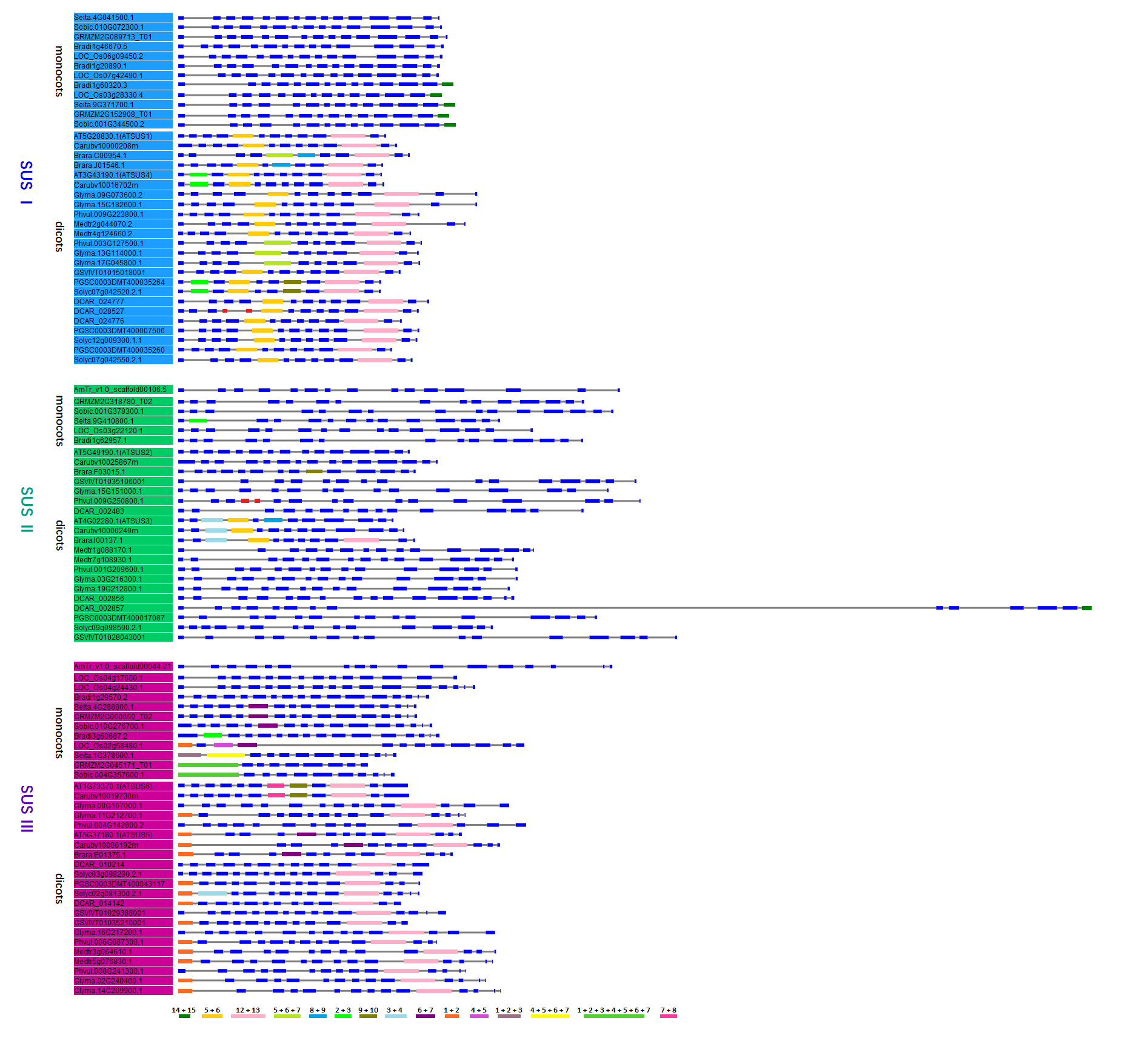

Supplement: Supplementary file 2 — Additional file 2: Figure S2. Intron/exon structural organization of angiosperms SUS genes. Blue boxes denote exons within coding regions, and the gray lines connecting them represent introns. Boxes of other colors represent fused exons, and the numbers above them indicate which exons are fused. Red boxes indicate the splitting of the exons. Due to the complexity of the 3′ end of the SUS III subfamily genes, we do not show the fusion of their exons. [file 12870_2019_2181_MOESM2_ESM.xlsx]

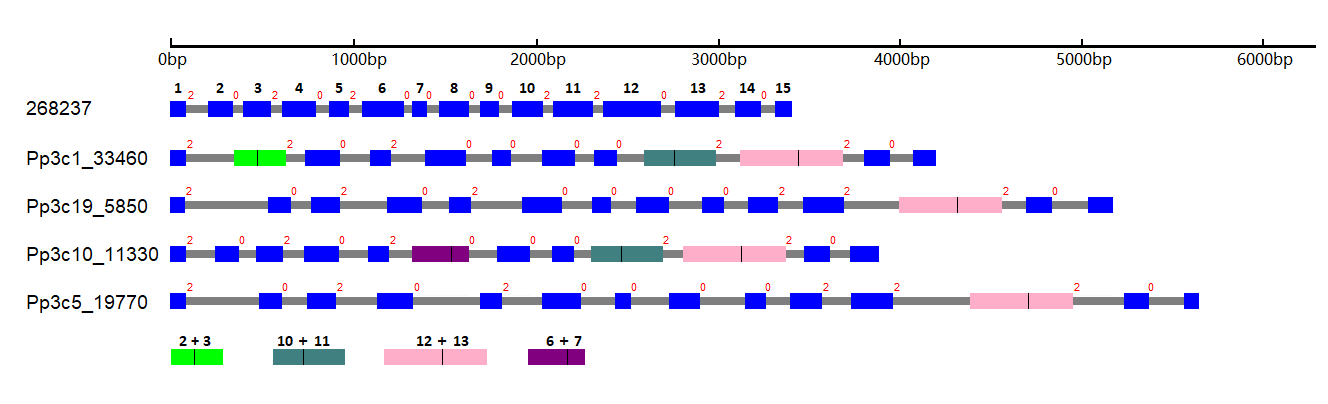

Supplement: Supplementary file 3 — Additional file 3: Figure S3. Intron/exon structural organization of SUS genes from P. patens and S. moellendorffii. Blue boxes denote exons within coding regions, and the gray lines connecting them represent introns. Red numbers on the upper left of the gray lines represent the intron phase of corresponding introns. Boxes of other colors represent fused exons, and the numbers above them indicate which exons are fused. [file 12870_2019_2181_MOESM3_ESM.tif]

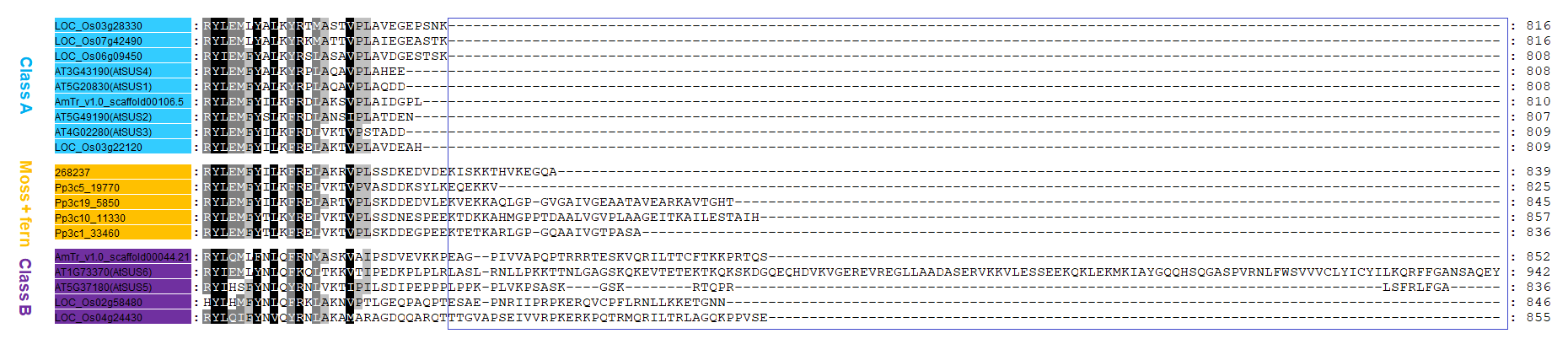

Supplement: Supplementary file 4 — Additional file 4: Figure S4. C-terminal amino acid alignment of SUS genes from A. thaliana, O. sativa, P. patens and S. moellendorffii. [file 12870_2019_2181_MOESM4_ESM.tif]

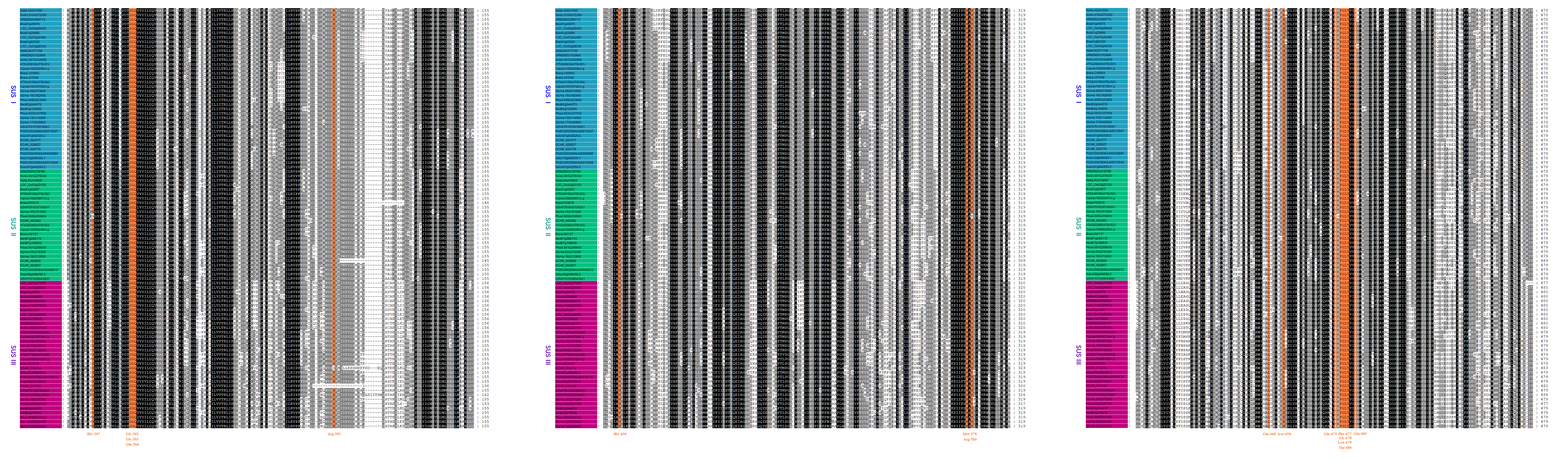

Supplement: Supplementary file 5 — Additional file 5: Figure S5. Amino acid alignment of GT-B domain of SUS genes. The 16 active sites (His-287, Gly-302, Gly-303, Gln-304, Arg-382, His-438, Met-578, Arg-580, Gln-648, Asn-654, Glu-675, Phe-677, Gly-678, Leu-679, Thr-680, Glu-683) identified in the GT-B domain of AtSUS1 were colored in orange. [file 12870_2019_2181_MOESM5_ESM.tif]
