# Supplementary material for: The evolutionary history of the sucrose synthase gene family in higher plants
Source: BMC Plant Biol. 2019 Dec 18;19:566. doi: 10.1186/s12870-019-2181-4 (PMC6921546; doi:10.1186/s12870-019-2181-4)
Supplement: Supplementary file 7 — Additional file 7: Table S2. Ks values of SUS gene pairs within subfamily of G. max. [file 12870_2019_2181_MOESM7_ESM.tif]

**Table S2.** ***Ks* values of SUS gene pairs within subfamily of *G. max*.**

| **Subfamily** | **Gene1** | **Gene2** | ***Ks*** |
| --- | --- | --- | --- |
|  | Glyma.13G114000.1 | Glyma.17G045800.1 | 0.1307 |
|  | Glyma.09G073600.1 | Glyma.15G182600.1 | 0.1308 |
|  | Glyma.09G073600.1 | Glyma.17G045800.1 | 0.5869 |
| **SUS I** | Glyma.15G182600.1 | Glyma.13G114000.1 | 0.5648 |
|  | Glyma.15G182600.1 | Glyma.17G045800.1 | 0.5410 |
|  | Glyma.09G073600.1 | Glyma.13G114000.1 | 0.5998 |
|  |  |  |  |
|  | Glyma.03G216300.1 | Glyma.19G212800.1 | 0.0671 |
| **SUS II** | Glyma.15G151000.1 | Glyma.03G216300.1 | 1.8479 |
|  | Glyma.15G151000.1 | Glyma.19G212800.1 | 1.8358 |
|  |  |  |  |
|  | Glyma.02G240400.1 | Glyma.14G209900.1 | 0.0927 |
|  | Glyma.09G167000.1 | Glyma.16G217200.1 | 0.0883 |
|  | Glyma.11G212700.1 | Glyma.02G240400.1 | 0.5181 |
|  | Glyma.11G212700.1 | Glyma.14G209900.1 | 0.5521 |
| **SUS III** | Glyma.09G167000.1 | Glyma.14G209900.1 | 1.6494 |
|  | Glyma.09G167000.1 | Glyma.02G240400.1 | 1.8246 |
|  | Glyma.16G217200.1 | Glyma.02G240400.1 | 1.8664 |
|  | Glyma.16G217200.1 | Glyma.14G209900.1 | 1.6405 |
|  | Glyma.09G167000.1 | Glyma.11G212700.1 | 1.6620 |
|  | Glyma.11G212700.1 | Glyma.16G217200.1 | 1.7488 |
