# Supplementary material for: The evolutionary history of the sucrose synthase gene family in higher plants
Source: BMC Plant Biol. 2019 Dec 18;19:566. doi: 10.1186/s12870-019-2181-4 (PMC6921546; doi:10.1186/s12870-019-2181-4)
Supplement: Supplementary file 10 — Additional file 10: Table S5. Expression patterns of Arabidopsis SUS genes under hypoxia. The expression data of six SUS genes in Arabidopsis comes from Gene Expression Omnibus (GEO) DataSets (GSE119327). We analyzed the expression of six SUS genes in Arabidopsis under hypoxia and found that only AtSUS1 and AtSUS4 were induced by hypoxia. adj. P.Val: P-value after adjustment for multiple testing. logFC: Log2-fold change between two experimental conditions. [file 12870_2019_2181_MOESM10_ESM.xlsx]

**Table S5.** **Expression patterns of *Arabidopsis* SUS genes under hypoxia.**

| **Subfamily** | **Gene** | **adj.P.Val** | **P.Value** | **logFC** |
| --- | --- | --- | --- | --- |
| **SUS I** | AT5G20830(*AtSUS1*) | 0.00026 | 5.15E-08 | 3.97 |
|  | AT3G43190(*AtSUS4*) | 0.00041 | 3.14E-07 | 5.94 |
|  |  |  |  |  |
| **SUS II** | AT5G49190(*AtSUS2*) | 0.40648 | 2.23E-01 | 0.31 |
|  | AT4G02280(*AtSUS3*) | 0.08645 | 1.80E-02 | 0.85 |
|  |  |  |  |  |
| **SUS III** | AT5G37180(*AtSUS5*) | 0.06321 | 1.09E-02 | -1.18 |
|  | AT1G73370(*AtSUS6*) | 0.45933 | 2.72E-01 | -0.24 |
